# Born in an Alien Nest : How Do Social Parasite Male Offspring Escape from Host Aggression?

**DOI:** 10.1371/journal.pone.0043053

**Published:** 2012-09-20

**Authors:** Patrick Lhomme, Manfred Ayasse, Irena Valterová, Thomas Lecocq, Pierre Rasmont

**Affiliations:** 1 Laboratory of Zoology, University of Mons, Mons, Belgium; 2 Institute of Experimental Ecology, Department of Chemical Ecology, University of Ulm, Ulm, Germany; 3 Institute of Organic Chemistry and Biochemistry, Academy of Sciences of the Czech Republic, Prague, Czech Republic; Université Paris 13, France

## Abstract

Social parasites exploit the colony resources of social insects. Some of them exploit the host colony as a food resource or as a shelter whereas other species also exploit the brood care behavior of their social host. Some of these species have even lost the worker caste and rely completely on the host's worker force to rear their offspring. To avoid host defenses and bypass their recognition code, these social parasites have developed several sophisticated chemical infiltration strategies. These infiltration strategies have been highly studied in several hymenopterans. Once a social parasite has successfully entered a host nest and integrated its social system, its emerging offspring still face the same challenge of avoiding host recognition. However, the strategy used by the offspring to survive within the host nest without being killed is still poorly documented. In cuckoo bumblebees, the parasite males completely lack the morphological and chemical adaptations to social parasitism that the females possess. Moreover, young parasite males exhibit an early production of species-specific cephalic secretions, used as sexual pheromones. Host workers might thus be able to recognize them. Here we used a bumblebee host-social parasite system to test the hypothesis that social parasite male offspring exhibit a chemical defense strategy to escape from host aggression during their intranidal life. Using behavioral assays, we showed that extracts from the heads of young cuckoo bumblebee males contain a repellent odor that prevents parasite males from being attacked by host workers. We also show that social parasitism reduces host worker aggressiveness and helps parasite offspring acceptance.

## Introduction

Parental investment strategies lie at the heart of fundamental life-history trade-offs [1], [2]. In many species, parents invest most of their energy in rearing their brood by producing a nest and providing it with protection and food. The high energetic costs of parental care have promoted the evolution of cheaters that exploit brood care behavior of conspecifics or heterospecifics. These brood parasites avoid the costs of parental care by laying their eggs in host nests. Their victims then care for the parasite offspring by incubating the eggs and feeding the progeny. Brood parasitism occurs in fishes, birds, amphibians [3]–[5], but is particularly well documented in social insects [6], [7].

In this last group, some parasites exploit the social behavior of their host and are so called social parasites. In social hymenopterans, some of these parasite species have even lost the worker caste and rely completely on the host worker's force to rear their offspring. These social parasites may possess several defensive morphological adaptations, such as a thickened cuticle and strengthened mandibles, which allow them to fight to enter nests successfully [8]–[10]. However the parasite females not only need to deter aggression, they also have to be adopted by host workers who then take care of the parasite offspring.

Social insects have evolved highly sophisticated recognition systems which enable them to behave altruistically towards relatives but to reject alien individuals, and therefore prevent their society's exploitation by parasites and predators [11]. These recognition systems consist of shared cuticular hydrocarbon profiles learned by all the members of a colony [12]. To avoid host defenses and bypass their recognition code, social parasites have therefore developed several chemical infiltration strategies. Some social parasites use a strategy called chemical insignificance, where they produce almost no detectable recognition cues on their cuticle [13]–[15]. A second strategy is chemical camouflage, where parasites after entering a host colony acquire these chemicals through direct contact with their hosts [16], [17]. Other species deceive the host through chemical mimicry. These species imitate host recognition cues by actively producing the host specific cuticular hydrocarbons [18]–[20]. Another strategy is the production of specific allomones that manipulate host behavior to the advantage of the parasite. These chemical cues include appeasement [21], [22], deterrent [23] or repellent [24]–[27] substances which protect parasite females from host aggressive behavior.

Infiltration strategies of social parasites have been intensively studied in several hymenopterans [6], [25], [28]–[30]. However, once the first line of defense has been broken, parasite's offspring still face the challenge of avoiding host recognition. Parasitized colonies have a reduced reproductive success [31], [32]. Hosts should therefore have evolved other defense tactics against social parasites such as recognition and rejection of parasite eggs and offspring. Moreover, in many cases the parasite female when entering a newly started host nests, needs to deceive only a few individuals, while the alien offspring has to fool a lot more host workers in order to survive in the nest. Some studies have already investigated the ability of social parasites in preventing their eggs from being destroyed by the host workers [33], [34], [35]. Cervo et al. [36] showed that social parasitic *Polistes* wasp larvae are able to overcome host recognition systems by producing a chemical blend that has no meaning to the host. Akino et al. [37] demonstrated that caterpillars of *Maculinea* butterflies are able to mimic the chemical profile of their host ants of the genus *Myrmica*. As a result, the host ants tolerate butterfly caterpillars in their brood chambers and continue to tend and feed them for months.

However, the strategy used by social parasite's adult offspring to survive within the host nest has been poorly studied.

In this study, we investigated a bumblebee host-social parasite system to test the hypothesis that social parasites have evolved an active strategy to escape from host recognition during their intranidal life. Cuckoo bumblebees (subgenus *Psithyrus*) have lost their ability to found their own nest [38]. These socially parasitic bumblebees all lack a worker caste and are thus completely dependent on their bumblebee hosts to rear their offspring. Once they have entered a host nest there appears to be a great variation among cuckoo bumblebees in their chemical infiltration strategies. Some inquiline bumblebees produce very low amounts of cuticular hydrocarbons and are able to acquire their host's chemical profile after intrusion [28], whereas others use repellents to defend themselves against the attacks of host workers [25]. Furthermore, in order to monopolize reproduction, parasite females selectively recognize and kill host workers with developed ovaries that would compete for male production [39] and are able to inhibit ovary development of the workers [30], [40].

It is assumed that young virgin females have the same strategy as their mothers because they share the same morphological and chemical adaptations to social parasitism. It is also known that freshly emerged bumblebees possess low amounts of cuticular lipids [39], [41]. Young parasite females may be tolerated in host colonies because they possess no recognition cues at emergence and later acquire the colony chemical signature through exchange of chemicals with members of the colony, as is the case of ants [42]. Cuckoo bumblebee males on the other hand don't have such a defensive potential as their mothers (sting or Dufour's gland producing repellent allomones). Moreover, males should not be able to escape from host recognition because of their early production of species specific cephalic secretions (see Supporting Information S1). In bumblebees, male cephalic secretions are mostly produced by the cephalic labial glands [43]. These pair glands produce in high quantity the male sexual marking pheromones [44] involved in the pre-mating behavior [45]. They are situated in the head, behind the eyes, and they occupy most of the head volume [46]. If parasite males fail to fool the recognition system of their host, they could still persist in the host nest provided they manage to avoid being rejected. We hypothesize that socially parasitic *Bombus* males therefore evolved a chemical defense strategy by producing allomones within their cephalic secretions to cheat the host workers and prevent rejection.

We tested this hypothesis by performing behavioral recognition assays: we tested whether the cephalic secretions of parasite males can elicit an aggressive response towards host workers. A single glass ball lure was presented during five minutes to four non-parasitized host colonies; some of the lures were coated with cephalic secretions of male parasites, while others were coated with cephalic secretions of conspecific males. We also tested whether the reactions of host workers towards parasite males are simply because they belong to a different species. Therefore, we also tested lures coated with cephalic secretions of a non-parasitic and phylogenetically distant species. We finally tested if social parasitism and parasite male presence modify workers discrimination ability and help parasite male's acceptance by performing the same experiment with four parasitized colonies and four parasitized colonies with emerged male parasites.

## Material and Methods

### Rearing bumblebees

The cuckoo bumblebee *Bombus vestalis vestalis* and its host *Bombus terrestris terrestris* were used as a model. *B. terrestris* colonies were reared by Biobest *bvba* from wild queens collected in the field near Westerlo, Belgium (51°05′23″N, 4°54′51″E, alt. 18 m). *B. vestalis* females were collected in spring in the surroundings of Mons, Belgium (50°28′21″N, 3°56′46″E, alt. 30 m). A third species, the non-parasitic and phylogenetically distant species *Bombus pascuorum floralis*, was used to test whether the reactions of host workers towards parasite males were simply because they belong to a different species. In spring, *B. pascuorum floralis* queens were collected near Westerlo, Belgium, transferred into wood nest boxes (28.5×13×12 cm) and used to produce males for cephalic secretions sampling. Females of *B. vestalis* are queen-intolerant inquilines that kill the host queen after colony usurpation. For these reasons, host queens were removed before the *Psithyrus* females were introduced into young *B. terrestris* colonies containing about fifteen workers immediately. All the species were reared in a dark room at 26–28°C and 65% humidity and fed *ad libitum* with sugar syrup (BIOGLUC®, Biobest) and fresh willow pollen (*Salix sp.*).

Males of *B. vestalis* and *B. terrestris* produced in the lab were collected to sample cephalic secretions. In total four parasitized colonies, four parasitized colonies with emerged male parasites and four non-parasitized queenright colonies of *B. terrestris* were used for behavioral assays.

### Extraction of bumblebee male cephalic secretions

De Meulemeester et al. [47] recently compared the quantitative and qualitative compositions of bumblebee male cephalic extracts with dissected cephalic labial glands and showed no difference between both extraction methods. Their method was thus used for male cephalic secretion extraction (e.g. [48]). Male cephalic secretions of *B. terrestris*, *B. vestalis*, and *B. pascuorum* were extracted from males of one colony of each species. Specimens were first killed by brief deep-freezing. They were then decapitated and each head was placed in a glass vial with 400 µl of hexane for compounds extraction. All samples were kept for 24 h at room temperature (20°C) to complete the extraction and then stored at −40°C until chemical analyses.

### Chemical Analyses

The cephalic secretions of five males of *B. vestalis* of two different ages (one day old and seven days old males) were analyzed to examine the early production of cephalic gland secretions. Cephalic secretions of males of *B. terrestris* and *B. pascuorum* were also analyzed to point out the differences between conspecific and heterospecific secretions. Samples were analyzed using a gas chromatograph Shimadzu GC-2010 with a SLB-5ms non-polar capillary column (5% diphenyl/95% dimethyl siloxane; 30 m×0.25 mm×0.25 µm) and a flame ionization detector. A splitless injector mode (220°C) and helium as carrier gas (50 cm/s) were used. The temperature program of the column was 70°C for 2 min, 10°C/min to 320°C and 5 min hold. The relative proportions in percentage of each compound were calculated by summing up the absolute amounts of all compounds using GCsolution Postrun (Shimadzu). The data matrix was created with the relative proportion of each compound for each individual. The identification of the compounds was performed using a gas chromatograph - mass spectrometer (GC-MS) Finnigan GCQ with a DB-5ms non-polar capillary column (5% phenyl methyl polysiloxane stationary phase; 30 m×0.25 mm×0.25 mm) and an ion trap in electron ionization mode (scan range : 30–600 amu). A splitless injector mode (220°C) and helium as carrier gas (50 cm/s) were used. The temperature program of the column was 70°C for 2 min, 10°C/min to 320°C and 5 min hold. Compounds were identified in Xcalibur™ using their mass spectra compared to those at National Institute of Standards and Technology library (NIST, U.S.A) using NIST MS Search 2.0.

### Behavioral experiments

In the first set of experiments, we tested whether the host workers were more aggressive towards conspecific male secretions than towards the parasite males' ones. The cephalic secretion samples used for behavioral assays were taken from a glass vial containing 400 µl of one head cephalic extract (one male equivalent). The glass ball lures (2 cm in diameter) were coated with 25 µl of the tested sample. We used two controls, lures without odor and lures coated with 25 µl of hexane (solvent). The odorant lure exposure was made under red light and video-recorded during five minutes. The videos were named with random codes so that the observer did not know the particular treatments. The observer noted the mean duration and the cumulative frequency of aggressive behavior (mid-leg raising, rolling on the back to present the sting and attacking, as described by Duchateau [49]) and the frequency of avoidance behavior that *B. terrestris* workers performed towards the glass balls. Retreat or rejection (stepping backward) were termed “avoidance behavior”.

We also tested if the parasite males' acceptance could be due to a decrease of worker recognition in parasitized colonies. We performed the same experiments on four queenless *B. terrestris* colonies parasitized by the social parasite *B. vestalis*. We finally tested whether the presence of parasite males can affect host worker recognition abilities. Therefore, we repeated the previous experiment on four queenless parasitized colonies of *B. terrestris* with emerged *B. vestalis* males.

All the experiments were performed using *B. terrestris* colonies of the same age (8–10 weeks after the first eggs were laid), with the same number of workers (n∼50) in plastic nest boxes of the same size (32×23×12 cm). We chose old colonies to be the closest possible of the social context of offspring emergence. Although old colonies were used, they were not old enough that they had entered the competition phase (no aggressive interactions between workers or between workers and queen were noticed). Before the experiment, all the glass ball lures were washed with solvent to remove any compounds. Treatments were performed one after the other on randomly chosen colonies and repeated every 24 h. Each treatment was repeated four times on each colony.

### Statistical analysis

To test the effect of the colony type (NP, P, PM) and the treatment (Co, Ht, Pa, S, T) on host worker behavior, data were compared by performing GLM (Poisson error, log link function). The colonies were nested in the colony types and treated as random variables. GLM was performed using an ANOVA and chi-square test as test criterion. *Post hoc* pair-wise comparisons were made using Tukey contrasts (multicomp package in R). The experiment was performed using cephalic secretions produced at two different ages for each species (one-day old and seven-day old males) to test the effect of male age on host worker behavioral response. However, we found no effect of male age in worker responses (frequency of aggressive behavior, duration of aggressive behavior and avoidance behavior) between ages (GLM-poisson, *p*>0.05, *p*>0.05, *p*>0.05). Thus, all data were pooled and treated together.

## Results

### Composition of the males' cephalic secretions of *B. vestalis*, *B. terrestris* and *B. pascuorum*

Amongst the compounds present in the secretions of *B. vestalis*, we identified hydrocarbons (*n*-alkanes and *n*-alkenes), aliphatic alcohols, aldehydes, esters, and isoprenoids (Table S1 in supporting information). These results clearly show that nearly all the compounds found in the cephalic glands in seven days old males are already produced in the early days of their life.

The qualitative and quantitative composition of the cephalic secretions of *B. vestalis*, *B. terrestris* and *B. pascuorum* clearly demonstrate the species-specificity of these secretions (Table S2 in supporting information). Among the 71 compounds identified, 48 are species-specific (16/30 in *B. terrestris*, 18/37 in *B. pascuorum* and 14/30 in *B. vestalis*). Only seven compounds are common to the three species, four alkanes : heneicosane (*n*-C_21_), tricosane (*n*-C_23_), pentacosane (*n*-C_25_) and heptacosane (*n*-C_27_); and three alkenes : pentacosene (*n*-C_25:1_), heptacosene (*n*-C_27:1_) and nonacosene (*n*-C_29:1_). Host and parasite share only 10 compounds, while they both share more with *B. pascuorum* (11 and 13 respectively, Table S1).

### Effect of parasite cephalic secretion extracts on worker behavior

**Figure 1:**
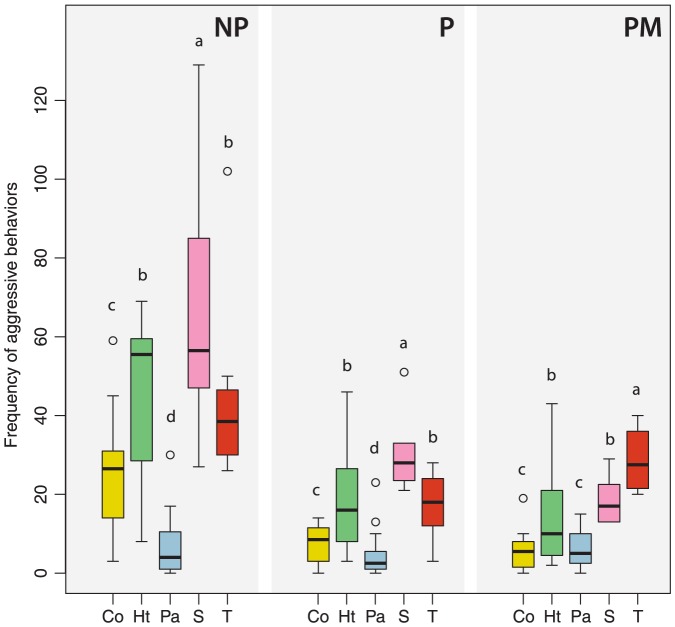
Frequency of host worker aggressive behaviors. Towards lures coated with cephalic secretions of conspecific males (Co), heterospecific males (Ht), parasite males (Pa) and controls (S = solvent, T = odorless). Treatments were performed during 5 minutes and repeated four times on four non-parasitized colonies (NP), four parasitized colonies (P) and four parasitized colonies with emerged males (PM). Box plots show the median and 25–75% percentiles. Whiskers show all data excluding outliers. Outliers (circles) are values being more than 1.5 times box length from upper and lower edge of respective box. The different letters indicate significant differences between treatments within experiments (GLM-poisson followed by Tukey's *post hoc* test, *p*<0.05).

**Figure 2:**
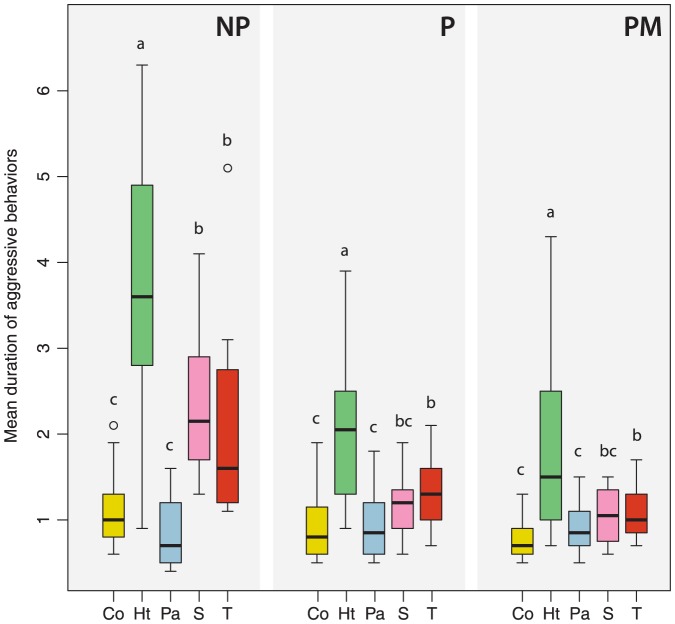
Mean duration (in seconds) of host worker aggressive behaviors. Towards lures coated with cephalic secretions of conspecific males (Co), heterospecific males (Ht), parasite males (Pa) and controls (S = solvent, T = odorless). Treatments were performed during 5 minutes and repeated four times on four non-parasitized colonies (NP), four parasitized colonies (P) and four parasitized colonies with emerged males (PM). Box plots show the median and 25–75% percentiles. Whiskers show all data excluding outliers. Outliers (circles) are values being more than 1.5 times box length from upper and lower edge of respective box. The different letters indicate significant differences between treatments within experiments (GLM-poisson followed by Tukey's *post hoc* test, *p*<0.05).

In non-parasitized colonies (NP), significant differences were found in the frequency and duration of host worker aggression between the different treatments (GLM-poisson; Df = 4, *p*<0.001; Df = 4, *p*<0.001, respectively). *B. terrestris* workers were significantly less aggressive towards the lures coated with cephalic extracts of parasite males (Pa) than towards the controls (S and T) (GLM-poisson, Tukey's tests, *p*<0.001 and *p*<0.001; Figure 1), lures coated with extracts of heterospecific males (Ht) (GLM-poisson, Tukey's test, *p*<0.001; Figure 1) and conspecific males (Co) (GLM-poisson, Tukey's test, *p*<0.001; Figure 1). The frequency of worker aggressive behaviors was not significantly different between lures coated with secretions of heterospecific males and the odorless control (T). The solvent control exhibited a higher rate of aggressive behavior than lures coated with secretions of heterospecific males (GLM-poisson, Tukey's test, *p*<0.001). But these three treatments (Ht, S, and T) induced a significantly higher frequency of worker aggressive behaviors compared to lures treated with conspecific cephalic secretions (GLM-poisson, Tukey's tests, *p*<0.001, *p*<0.001 and *p*<0.001; Figure 1). Moreover, these three treatments (Ht, S and T) induced a significantly longer duration of host worker aggressive behaviors compared to lures coated with secretions of conspecific males (GLM-poisson, Tukey's tests, *p*<0.001, *p*<0.001 and *p*<0.01; Figure 2) or parasite males (GLM-poisson, Tukey's tests, *p*<0.001, *p*<0.001 and *p*<0.001; Figure 2). The duration of worker aggressive behaviors was not significantly different between the controls.

**Figure 3:**
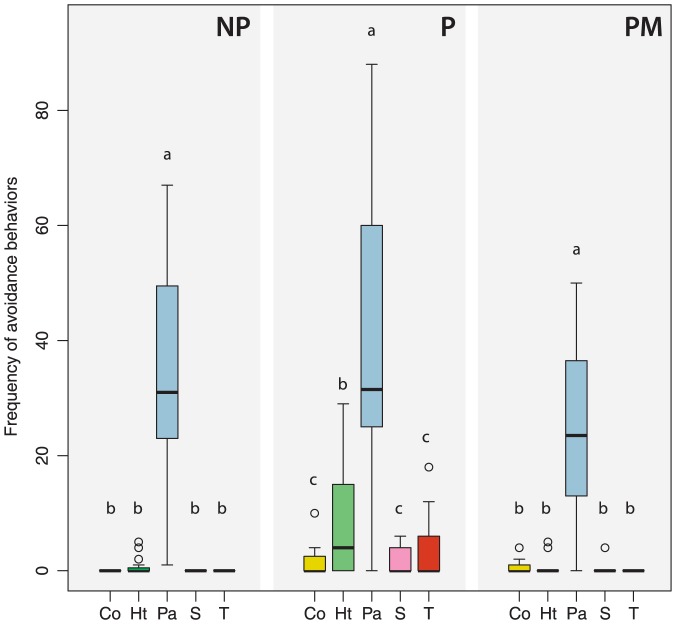
Frequency of host worker avoidance behaviors. Towards lures coated with cephalic secretions of conspecific males (Co), heterospecific males (Ht), parasite males (Pa) and controls (S = solvent, T = odorless). Treatments were performed during 5 minutes and repeated four times on four non-parasitized colonies (NP), four parasitized colonies (P) and four parasitized colonies with emerged males (PM). Box plots show the median and 25–75% percentiles. Whiskers show all data excluding outliers. Outliers (circles) are values being more than 1.5 times box length from upper and lower edge of respective box. The different letters indicate significant differences between treatments within experiments (GLM-poisson followed by Tukey's *post hoc* test, *p*<0.05).

We also found significant differences in the frequency of host worker avoidance behavior between the different treatments (GLM-poisson, Df = 4, *p*<0.001). Lures coated with secretions of parasite males (Pa) were also significantly more avoided than the controls (S and T) (GLM, Tukey's tests, *p*<0.001 and *p*<0.001; Figure 3) and the lures coated with extracts of conspecific males (GLM-poisson, Tukey's test, *p*<0.001; Figure 3) and heterospecific males (Tukey's HSD test, *p*<0.001; Figure 3). All the other treatments (Co, Ht, S, T) did not show significant difference in the frequency of worker avoidance behavior.

### Effect of social parasitism on worker behavior

We found the same pattern of worker response between the different lures in parasitized colonies (P) and in non-parasitized colonies (NP). Nevertheless, the host workers exhibited a significantly lower frequency of aggressive behaviors in parasitized colonies (GLM-poisson, Tukey's test, *p*<0.001; Figure 1) and the mean duration of the aggressive behaviors was shorter (GLM-poisson, Tukey's test, *p*<0.05; Figure 2). We found significant differences in the frequency and duration of host worker aggression between the different treatments in parasitized colonies (GLM-poisson, *p*<0.001). The frequency of worker aggressive behaviors was significantly lower towards lures coated with parasite secretions compared to lures coated with conspecific secretions (GLM-poisson, Tukey's test, *p*<0.05), heterospecific secretions (GLM-poisson, Tukey's test, *p*<0.001) or compared to the controls (GLM-poisson, Tukey's test, *p*<0.05; Figure 1). Lures coated with cephalic secretions of conspecific males were significantly less subjected to aggressive behaviors than the controls (S and T) (GLM-poisson, Tukey's tests, *p*<0.001 and *p*<0.001; Figure 1) or the lures coated with secretions of heterospecific males (GLM-poisson, Tukey's test, *p*<0.001; Figure 1). Cephalic secretions of heterospecific males induced a longer duration of aggressive behaviors compared to the secretions of conspecific males (GLM-poisson, Tukey's test, *p*<0.001; Figure 2), parasite males (GLM-poisson, Tukey's test, *p*<0.001; Figure 2) and the solvents (S and T) (GLM-poisson, Tukey's test, *p*<0.001, *p*<0.01; Figure 2).

Finally, we found no difference in host worker avoidance behaviors between parasitized colonies (P) and non-parasitized colonies (NP). However, there were still significant differences in the frequency of host worker avoidance behaviors between the different treatments. The secretions of parasite males were significantly more avoided than the other lures (Co, Ht, S and T) (GLM-poisson, Tukey's tests, *p*<0.001, *p*<0.001, *p*<0.001 and *p*<0.01; Figure 3).

### Effect of the presence of parasite males on worker behavior

We found no difference in the frequency and duration if host worker aggressive behavior between the three colony types. We found, however, significant differences in host worker avoiding behavior (GLM-poisson, Tukey's tests, P-PM: *p*<0.001; NP-PM: *p*<0.001). Compared to non-parasitized colonies, host workers also exhibited a significant decrease in the frequency of aggressive behaviors (GLM-poisson, Tukey's test, *p*<0.001; Figure 1) in parasitized colonies with emerged males. The mean duration of the aggressive behaviors was also shorter (GLM-poisson, Tukey's test, *p*<0.01; Figure 2). Nevertheless, there were significant differences in the frequency and duration of host worker aggressive behaviors between the different treatments (GLM-poisson, Df = 4, *p*<0.001). Workers in presence of emerged *B. vestalis* males exhibited higher frequency of aggressive behaviors when confronted with lures coated with cephalic secretions of heterospecific males in comparison to lures coated with secretions of conspecific males (GLM-poisson, Tukey's tests, *p*<0.001; Figure 1) or parasite males (GLM-poisson, Tukey's tests, *p*<0.001; Figure 1). They also exhibited a longer duration of aggressive behaviors (GLM-poisson, Tukey's tests, *p*<0.001 and *p*<0.001; Figure 2).

We also found significant differences in the frequency of host worker avoidance behaviors between the different treatments in (GLM-poisson, Df = 4, *p*<0.001). The secretions of parasite males were significantly more avoided than the other lures (Co, Ht, S and T) (GLM-poisson, Tukey's tests, *p*<0.001, *p*<0.001, *p*<0.001 and *p*<0.001; Figure 3).

## Discussion

### Parasite cephalic secretions and host worker behavior

The results of our odor presentation experiments first clearly demonstrate that host workers can recognize and discriminate against heterospecifics on the basis of the cephalic secretions of males. *Bombus terrestris* workers show higher frequency and duration of aggression behaviors towards lures coated with chemical cues produced by heterospecific males (Figures 1 and 2). As has already been described in literature [50]–[52], we show that each species produces a species-specific blend of chemical cues mostly consisting of hydrocarbons, aliphatic alcohols, aldehydes, esters and isoprenoids. It is known that hydrocarbons do play a central role in the nestmate recognition system of bumblebees [20]. Therefore the blend of hydrocarbons may thus encode the information on species identity that enables *B. terrestris* workers to recognize alien species. But we cannot exclude that the other compounds secreted play also a role in worker discrimination. Several studies have already demonstrated the importance of non-cuticular hydrocarbons in nestmate discrimination. In two ant species of the genus *Atta*, discrimination cues seem to be mediated both by alarm pheromones and abdominal exocrine secretions [53]. Another study suggested that volatiles could act as short distance discrimination cues in *Lasius fuliginosus* [54]. Finally in *Camponotus fellah*, volatiles secreted by the Dufour's gland have been shown to be involved in nestmate recognition [55], [56].

To our surprise, both controls are generally more attacked by the host workers than lures coated with cephalic secretions. The repeated exposure to cephalic gland extracts could have induced a decrease of aggressiveness due to a phenomenon of odor habituation. However, the high frequency of aggression against the controls was exhibited since the beginning of the experiments, before odor habituation could arise. Moreover, the mean duration of host worker aggressive behavior towards the controls tends to be shorter compared to lures coated with heterospecific secretions. The workers seem to display a different pattern of aggressive behavior towards the controls. It is more likely that the simple intrusion of a foreign object into the colony is enough to engender aggressions.

The results of our first experiment (Figure 3) also show a clear repellent effect of the male cephalic secretions of *B. vestalis* males on *B. terrestris* workers. The cephalic extract of parasite males induces a high frequency of retreats and consequently reduced aggression of host workers towards the lures. This suggests that *B. vestalis* males may escape from host aggression by producing odorants that repel *B. terrestris* host workers. Several social parasites use repellent allomones as an adaptation to avoid host worker aggression during invasion of host colonies. This strategy is quite common in ants [22], [24], [26], [57] but also in bumblebees. *Bombus norvegicus* females secrete dodecyl acetate from their enlarged Dufour's gland as a chemical repellent [25]. Martin et al. [20] recently showed that females of the social parasites *Bombus sylvestris*, *B. bohemicus* and *B. vestalis* also produce this worker repellent allomone from their Dufour's gland. To our knowledge, there is only one example of a role of cephalic secretions of bee males in a host-parasite interaction. A co-occurrence of the major chemical compounds has been found in the cephalic secretions of parasite males of *Nomada* species and in Dufour's glands of their hosts of genus *Melitta* and *Andrena*. Males of the parasite apparently transfer their cephalic secretions to the female during mating, and the persisting compounds then assist the female in penetrating the host nest, presumably by mimicking host Dufour's gland odor [58], [59]. In this case, the male cephalic secretion enables the infiltration of the parasite female into the host nest.

The two major components of the cephalic secretions of *B. vestalis* males are geranylcitronellyl acetate and geranylcitronellol ([51], [60], Table S1). These compounds are not known to have a particular repellent effect in bumblebees and are probably involved in sexual attraction [50], [61]. Three other compounds, octadecenol, hexadecenyl acetate and tetradecyl acetate are present in the repellent secretions of *B. norvegicus* [25] and could thus play the same role in *B. vestalis* cephalic secretions. However octadecenol and hexadecenyl acetate are also present in the cephalic secretions of *B. pascuorum*. Both should therefore not be the main compounds with a function as repellent allomone. In contrast, tetradecyl acetate is only present in the secretions of *B. vestalis* and is also known as a common molecule in the defensive secretions of several arthropods [62]. Further studies will be needed to identify specific compounds involved in this allomonal effect.

### Social parasitism and host worker behavior

The results of our second experiment shows that host workers exhibit a diminishing degree of aggression in parasitized colonies compared to non-parasitized ones. This suggests that social parasitism reduces host worker aggressiveness. Sramkova and Ayasse [39] recently demonstrated that *B. vestalis* females are able to selectively kill older host workers that directly compete with them for reproduction. The old workers are more likely to possess developed ovaries and are among the first egg-layers [63], [64]. They are the dominant and also the most aggressive ones. The *Psithyrus* females use this worker discrimination ability to maintain reproductive dominance and maximize their reproductive success. This strategy could also explain the reduced aggressiveness of parasitized colony.

These results could also be explained by a modification of the host colony odor due to social parasite presence. Colony social structure influences worker abilities to discriminate between nestmates and non-nestmates [65]. Investigations using mixed ant species groups of *Manica rubida* and *Formica selysi* demonstrated that *M. rubida* ants living in heterospecific groups exhibit a diminishing degree of aggression towards alien secretions. This has been explained by a process of habituation to the odor of *F. selysi* [66]. The tolerance observed in mixed-species groups is also attributed to a mutual modification of the recognition odor through acquisition of the heterospecific cuticular hydrocarbons, thus creating a common mixed profile [67]–[69].

This chemical plasticity is also involved in parasitic associations of ants, bees and wasps [28], [70], [71]. Social parasites are able to acquire conspecific host odors but also to add their own recognition cues to the host colony profile [72]. As in mixed-species groups, odors of social parasites may thus influence nestmate discrimination [73]–[75]. Lorenzi [76] has shown in *Polistes* wasps that the increased complexity of colony-profile in parasitized colonies affects the ability of host workers to distinguish between nestmates and non-nestmates. It suggests that parasite-specific compounds modify host colony odor and thus force the workers to update the learned template to a new one that incorporates the heterospecific compounds. The presence of *B. vestalis* females could thus result in a decrease of host worker recognition abilities that could facilitate acceptance of the parasite's offspring.

The decrease in host worker aggressiveness could also be a consequence of the absence of the host queen. Some studies, particularly in ants, suggest that workers in queenless nests are less aggressive towards intruders [77] and exhibit weaker discriminatory behavior [78], [79] than workers in queenright nests. It is generally assumed that the queen emits a primer pheromone that increases the discrimination threshold of workers. Recent studies also showed in honey bees and bumblebees that queenless colonies are more susceptible to invading alien workers [80]–[82], possibly due to a relaxation of the nestmate recognition system. However several investigations have also demonstrated that queens contribute little or nothing to worker nestmate discrimination abilities [83]–[86]. The queen's presence is not always the dominant factor influencing worker aggression towards alien individuals and seems probably to be more important in small colonies or in the early phase of colony development [86]. Moreover, Bloch et al. [87] demonstrated in *B. terrestris* that queenless workers have significantly more developed ovaries and overt aggression. But this was not the case in our experiments as workers in parasitized colonies were less aggressive than in non-parasitized colonies. Cuckoo bumblebee females not only usurp the resident queen's reproductive position but also manipulate host workers. They are able to suppress host worker ovarian development and to actively produce the host queen's fertility signals [30], [40]. Therefore parasitized colonies should not behave like queenless colonies.

### Presence of male parasites and host worker behavior

In the colonies with emerged male parasites, workers exhibited the same pattern of aggressiveness than workers in parasitized colonies without males. However, we showed that the frequency of avoidance behavior decreased in colonies with emerged male parasites, compared to the other types of colonies. We could hypothesize that the continuous presence of parasite male chemical cues induces a phenomenon of habituation of parasite male odor. Habituation is often advanced to explain interspecific cohabitation between ants [88]–[91] or in social parasitic associations when the interacting species have distinct chemical profiles [92].

In conclusion, we show that after emergence, males of the cuckoo bumblebee *B. vestalis* seem to produce a repellent odor that reduces host worker attacks during their intranidal life. We also demonstrate that social parasitism decreases worker aggressiveness towards alien individuals, possibly because of a decrease of host worker discrimination threshold. In addition to a chemical protection, male parasite offspring seem to be born in a facilitating social context that favors their acceptance by the host workers.

## Supporting Information

Table S1 **Relative abundance (median, rel. %) of the compounds identified in cephalic secretions of** ***Bombus vestalis*** **males.**
(DOC)

Table S2 **Relative abundance (median, rel. %) of the compounds identified in the cephalic secretions of the males of** ***Bombus terrestris*** **(** ***n*** **= 5),** ***B. pascuorum*** **(** ***n*** **= 12) and** ***B. vestalis*** **(** ***n*** **= 5).**
(DOC)

## References

[1] Clutton-Brock TH (1991). The evolution of parental care. Princeton: Princeton University Press. 368 p.

[2] Stearns SC (1992) The Evolution of Life Histories. Oxford: Oxford University Press. 248 p.

[3] Summers K, Amos W (1997) Behavioral, ecological, and molecular genetic analyses of reproductive strategies in the Amazonian dart-poison frog, *Dendrobates ventrimaculatus* . Behav Ecol 8: 260–267.

[4] Wisenden BD (1999) Alloparental care in fishes. Rev Fish Biol Fisher 9: 45–70.

[5] Davies NB (2000) Cuckoos, Cowbirds and Other Cheats. London: T and AD Poyser. 310 p.

[6] Lenoir A, D'Ettorre P, Errard C, Hefetz A (2001) Chemical ecology and social parasitism in ants. Annu Rev Entomol 46: 573–599. 11112180 10.1146/annurev.ento.46.1.573

[7] Nash DR, Boomsma JJ (2008) Communication between hosts and social parasites. In: Sociobiology of communication: an interdisciplinary perspective. HughesDP: Oxford University Press. pp. 55–79.

[8] Fisher RM, Sampson BJ (1992) Morphological specializations of the bumble bee social parasite *Psithyrus ashtoni* (Hymenoptera, Apidae). Can Entomol 124: 69–77.

[9] Ondricek-Fallscheer RL (1992) A morphological comparison of the sting apparatuses of socially parasitic and nonparasitic species of yellowjackets (Hymenoptera:Vespidae). Sociobiology 20: 245–300.

[10] Cervo R (1994) Morphological adaptations to the parasitic life in *Polistes sulcifer* and *Polistes atrimandibularis* (Hymenoptera, Vespidae). Ethol Ecol Evol Special Issue 3: 61–66.

[11] Wilson EO (1971) The Insect Societies. Cambridge: Harvard University Press. 562 p.

[12] Howard W, Blomquist GJ (2005) Ecological, behavioral, and biochemical aspects of insect hydrocarbons. Annu Rev Entomol 50: 371–393. 15355247 10.1146/annurev.ento.50.071803.130359

[13] D'Ettorre P, Errard C (1998) Chemical disguise during colony founding in the dulotic ant *Polyergus rufescens* Latr. (Hymenoptera, Formicidae). Insect Soc Life 2: 71–77.

[14] Johnson CA, Vander Meer RK, Lavine B (2001) Changes in the cuticular hydrocarbon profile of the slave-maker ant queen, *Polyergus breviceps* Emery, after killing a Formica host queen (Hymenoptera: Formicidae). J Chem Ecol 27: 1787–1804. 11545371 10.1023/a:1010456608626

[15] Lambardi D, Dani FR, Turillazzi S, Boomsma JJ (2007) Chemical mimicry in an incipient leaf-cutting ant social parasite. Behav Ecol Sociobiol 61: 843–851.

[16] Dettner K, Liepert C (1994) Chemical mimicry and camouflage. Annu Rev Entomol 39: 129–154.

[17] Lenoir A, Cuisset D, Hefetz A (2001) Effects of social isolation on hydrocarbon pattern and nestmate recognition in the ant *Aphaenogaster senilis* (Hymenoptera, Formicidae). Insect Soc 48: 101–109.

[18] Howard RW, McDaniel CA, Nelson DR, Blomquist GJ, Gelbaum LT, et al (1982) Cuticular hydrocarbons of *Reticulitermes virginicus* (Banks) and their role as potential species- and caste recognition cues. J Chem Ecol 8: 1227–1239. 24413965 10.1007/BF00990755

[19] Bauer S, Witte V, Böhm M, Foitzik S (2009) Fight or flight? A geographic mosaic in host reaction and potency of a chemical weapon in the social parasite *Harpagoxenus sublaevis* . Behav Ecol Sociobiol 64: 45–56.

[20] Martin SJ, Carruthers JM, Williams PH, Drijfhout FP (2010) Host specific social parasites (*Psithyrus*) reveal evolution of chemical recognition system in bumblebees. J Chem Ecol 36: 855–863. 20509042 10.1007/s10886-010-9805-3

[21] Topoff H, Cover S, Greenburg L, Goodloe L, Sherman P (1988) Colony founding by queens of the obligatory slave-making ant *Polyergus breviceps*: The role of the Dufour's gland. Ethology 78: 209–218.

[22] Mori A, Grasso DA, Visicchio R, Le Moli F (2000) Colony founding in *Polyergus rufescens*: the role of the Dufour's gland. Insect Soc 47: 7–10.

[23] Martin SJ, Jenner EA, Drijfhout FP (2007) Chemical deterrent enables a socially parasitic ant to invade multiple hosts. P Roy Soc B-Biol Sci 274: 2717–2722. 10.1098/rspb.2007.0795 PMC2279212 17711838

[24] D'Ettorre P, Errard C, Ibarra F, Francke W, Hefetz A (2000) Sneak in or repel your enemy: Dufour's gland repellent as a strategy for successful usurpation in the slave-maker *Polyergus rufescens* . Chemoecology 10: 135–142.

[25] Zimma BO, Ayasse M, Tengö J, Ibarra F, Schultz C, et al (2003) Do social parasitic bumblebees use chemical weapons? J Comp Physiol A 189: 769–775. 10.1007/s00359-003-0451-x 12955437

[26] Ruano F, Hefez A, Lenoir A, Francke W, Tinaut A (2005) Dufour's gland secretion as a repellent used during usurpation by the slave-maker ant *Rossomyrmex minuchae* . J Insect Physiol 51: 1158–1164. 16076474 10.1016/j.jinsphys.2005.06.005

[27] Tsuneoka Y, Akino T (2009) Repellent effect on host Formica workers of queen Dufour's gland secretion of the obligatory social parasite ant, *Polyergus samurai* (Hymenoptera: Formicidae). Appl Entomol Zool 44: 133–141.

[28] Dronnet S, Simon X, Verhaeghe JC, Rasmont P, Errard C (2005) Bumblebee inquinilism in *Bombus (Fernaldaepsithyrus) sylvestris* (Hymenoptera, Apidae) : behavioural and chemical analyses of host-parasite interactions. Apidologie 36: 59–70.

[29] Cervo R (2006) *Polistes* wasps and their social parasites: an overview. Ann Zool Fenn 43: 531–549.

[30] Kreuter K, Bunk E, Lückemeyer A, Twele R, Francke W, et al (2011) How the social parasitic bumblebee *Bombus bohemicus* sneaks into power of reproduction. Behav Ecol Sociobiol 66: 475–486.

[31] Fisher RM (1987) Queen-worker conflict and social parasitism in bumblebees (Hymenoptera: Apidae). Anim Behav 35: 1026–1036.

[32] Lorenzi MC, Cervo R, Turillazzi S (1992) Effects of social parasitism of *Polistes atrimandibularis* on the colony cycle and brood production of *Polistes biglumis bimaculatus* (Hymenoptera, Vespidae). Boll Zool 59: 267–271.

[33] Johnson CA, Topoff H, van der Meer RK, Lavine B (2005) Do these eggs smell funny to you? : an experimental study of egg discrimination by hosts of the social parasite *Polyergus breviceps* (Hymenoptera: Formicidae). Behav Ecol Sociobiol 57: 245–255.

[34] Martin SJ, Takahashi J, Ono M, Drijfhout FP (2008) Is the social parasite *Vespa dybowskii* using chemical transparency to get her eggs accepted? J Insect Physiol 54: 700–707. 18342329 10.1016/j.jinsphys.2008.01.010

[35] Chernenko A, Helanterä H, Sundström L (2011) Egg Recognition and Social Parasitism in Formica Ants. Ethology 117: 1081–1092.

[36] Cervo R, Dani FR, Cotoneschi C, Scala C, Lotti I, et al (2008) Why are larvae of the social parasite wasp *Polistes sulcifer* not removed from the host nest? Behav Ecol Sociobiol 62: 1319–1331.

[37] Akino T, Knapp JJ, Thomas JA, Elmes GW (1999) Chemical mimicry and host specificity in the butterfly *Maculinea rebeli*, a social parasite of *Myrmica* ant colonies. Proc R Soc Lond B 266: 1419–1426.

[38] Benton T (2006) Bumblebees. London: Harper Collins. 580 p.

[39] Sramkova A, Ayasse M (2009) Chemical ecology involved in invasion success of the cuckoo bumblebee *Bombus vestalis* and workers of its host *Bombus terrestris* . Chemoecology 19: 55–62.

[40] Vergara CH, Schröder S, Almanza MT, Wittmann D (2003) Suppression of ovarian development of *Bombus terrestris* workers by *B. terrestris* queens, *Psithyrus vestalis* and *Psithyrus bohemicus* females. Apidologie 34: 563–568.

[41] Ayasse M, Marlovits TC, Tengö J, Taghizadeh T, Francke W (1995) Are there pheromonal dominance signals in the bumblebee *Bombus hypnorum* L (Hymenoptera, Apidae). Apidologie 26: 163–180.

[42] Soroker V, Vienne C, Nowbahari E, Hefetz A (1994) The Postpharyngeal gland as a “Gestalt” organ for nestmate recognition in the ant *Cataglyphis niger* . Naturwissenschaften 81: 510–513.

[43] Bergström G (1981) Chemical aspects of insect exocrine signals as a means for systematic and phylogenetic discussions in aculeate Hymenoptera. Entomol Scand 15: 173–184.

[44] Kullenberg B, Bergström G, Bringer B, Carlberg B, Cederberg B (1973) Observations on Scent Marking by *Bombus* Latr. and *Psithyrus* Lep. Males (Hym., Apidae) and Localization of Site of Production of the Secretion. Zoon Suppl. 1: 23–30.

[45] Bergman P (1997) Chemical communication in bumblebee premating behaviour. Ph-D thesis. Department of Chemical Ecology, Göteborg University, 26p

[46] Ågren L, Cederberg B, Svensson BG (1979) Changes with age in ultrastructure and pheromone content of male labial glands in some bumble bee species (Hymenoptera, Apidae). Zoon 7: 1–14.

[47] DeMeulemeester T, Gerbaux P, Boulvin M, Coppée A, Rasmont P (2011) A simplified protocol for bumble bee species identification by cephalic secretion analysis. Insect Soc 58: 227–236.

[48] Lecocq T, Lhomme P, Michez D, Dellicour S, Valterová I, et al (2011) Molecular and chemical characters to evaluate species status of two cuckoo bumblebees: *Bombus barbutellus* and *Bombus maxillosus* (Hymenoptera, Apidae, Bombini). Sys Ent 36: 453–469.

[49] Duchateau MJ (1989) Agonistic behaviours in colonies of the bumblebee *Bombus terrrestris* . J Ethol 7: 141–151.

[50] Coppée A, Mathy T, Cammaerts M-C, Verheggen FJ, Terzo M, et al (2011) Age-dependent attractivity of males' sexual pheromones in *Bombus terrestris* (L.) [Hymenoptera, Apidae]. Chemoecology 21: 75–82.

[51] Valterová I, Svatos A, Hovorka O (1996) Analysis of the labial gland secretion of the cuckoo-bumblebee (*Psithyrus vestalis*) males and synthesis of abundant geranylcitronellol. Collect Czech Chem Commun 61 1501–1508.

[52] Descoins C, Frerot B, Gallois M, Lettere M, Bergström G, et al (1984) Identification of compounds of the marking phormone produced by the labial glands of males of *Megabombus pascuorum* (Hymenoptera, Apidae). Nova Acta Regiae Soc Sci Upsal Ser V 3: 149–152.

[53] Hernandez JV, Goitia W, Osio A, Cabrera A, Lopez H, et al (2006) Leaf-cutter ant species (Hymenoptera: *Atta*) differ in the types of cues used to differentiate between self and others. Anim Behav 71: 945–952.

[54] Akino T, Yamaoka R (2000) Evidence for volatile and contact signals of nestmate recognition in the black shining ant *Lasius fuliginosus* Latreille (Hymenoptera: Formicidae). J Entomol Sc 3: 1–8.

[55] Katzav-Gozansky T, Boulay R, Vander Meer R, Hefetz A (2004) Innest environment modulates nestmate recognition in the ant *Camponotus fellah* . Naturwissenschaften 91: 186–190. 15085277 10.1007/s00114-004-0513-0

[56] Katzav-Gozansky T, Boulay R, Ionescu-Hirsh A, Hefetz A (2008) Nest volatiles as modulators of nestmate recognition in the ant *Camponotus fellah* . J Insect Physiol 54: 378–385. 18045612 10.1016/j.jinsphys.2007.10.008

[57] Visicchio R, Sledge MF, Mori A, Grasso DA, Le Moli F, et al (2000) Dufour's gland contents of queens of the slave-making ant *Polyergus rufescens* and its host species *Formica cunicularia* . Ethol Ecol Evol 12: 67–73.

[58] Tengö J, Bergström G (1976) Odor correspondence between *Mellita* females and males of their nest parasite *Nomada flavopicta* K. (Hymenoptera: Apoidea). J Chem Ecol 2: 57–65.

[59] Tengö J, Bergström G (1977) Cleptoparasitism and odor mimetism in bees: do *Nomada* males imitate the odor of *Andrena* females. Science 196: 1117–1119. 17778551 10.1126/science.196.4294.1117

[60] Urbanová K, Halík J, Hovorka O, Kindl J, Valterová I (2004) Marking pheromones of the cuckoo bumblebee males (Hym, Apoidea, *Bombus*): compositions of labial gland secretions of six species found in the Czech Republic. Biochem Sys Ecol 32: 1025–1045.

[61] Hovorka O, Urbanová K, Valterová I (1998) Premating behavior of *Bombus confusus* males and analysis of their labial gland secretion. J Chem Ecol 24: 183–193.

[62] Hefetz A, Lloyd A, Valdenbero A (1984) The defensive secretion of the tiger beetle *Cicindela flexuosa* (F.) (Cicindelinae; Carabidae). Experientia 40: 539–530.

[63] Honk CGJ van, Röseler PF, Velthuis HHW, Hoogeveen JC (1981) Factors influencing the egg laying of workers in a captive *Bombus terrestris* colony. Behav Ecol Sociobiol 9: 9–14.

[64] Duchateau MJ (1989) Ovarian development and egg laying in workers of *Bombus terrrestris* . Entomol Exp Appl 51: 199–213.

[65] Vander Meer RK, Morel L (1998) Nestmate recognition in ants. In: Vander Meer RK, Breed M, Winston M, Espelie KE, eds. Pheromone communication in social insects. Boulder: Westview. pp 79–103.

[66] Errard C, Hefetz A (1997) Label familiarity and discriminatory ability of ants reared in mixed groups. Insect Soc 44: 189–198.

[67] Hefetz A, Errard C, Cojocaru M (1992) The occurrence of heterospecific substances in the postpharyngeal gland secretion of ants reared in mixed species colonies (Hymenoptera: Formicidae). Naturwissenschaften 79: 417–420.

[68] Errard C (1994) Development of interpecific recognition behavior in the ants *Manica rubida* and *Formica selysi* (Hymenoptera: Formicidae) reared in mixed-species groups. J Insect Behav 7: 83–99.

[69] Vienne C, Soroker V, Hefetz A (1995) Congruency of hydrocarbon patterns in heterospecific groups of ants: transfer and/or biosynthesis? Insect Soc 42: 267–277.

[70] d'Ettorre P, Mondy N, Lenoir A, Errard C (2002) Blending in with the crowd: social parasites integrate into their host colonies using a flexible chemical signature. Proc R Soc Lond B Biol Sci 269: 1911–1918. 10.1098/rspb.2002.2110 PMC1691110 12350253

[71] Lorenzi MC, Caldi M, Cervo R (2007) The chemical strategies used by *Polistes nimphus* social wasp usurpers (Hymenoptera Vespidae). Biol J Linn Soc 91: 505–512.

[72] Lorenzi MC, Bagnères AG (2002) Concealing identity and mimicking hosts: a dual chemical strategy for a single social parasite? (*Polistes atrimandibularis*, Hymenoptera: Vespidae). Parasitology 125: 507–512. 12553569 10.1017/s003118200200238x

[73] Bagnères AG, Lorenzi MC, Clément JL, Dusticier G, Turillazzi S (1996) Chemical usurpation of a nest by paper wasp parasites. Science 272: 889–892. 8662579 10.1126/science.272.5263.889

[74] Turillazzi S, Sledge MF, Dani FR, Cervo R, Massolo A, et al (2000) Social hackers: integration in the host chemical recognition system by a paper wasp social parasite. Naturwissenschaften 87: 172–176. 10840803 10.1007/s001140050697

[75] Lorenzi MC, Cometto I, Marchisio G (1999) Species and colony components in the recognition odor of young social wasps: their expression and learning (*Polistes biglumis* and *P. atrimandibularis*; Hymenoptera: Vespidae). J Insect Behav 12: 147–158.

[76] Lorenzi MC (2003) Social wasp parasites affect the nestmate recognition abilities of their hosts (*Polistes atrimandibularis* and *P. biglumis*, Hymenoptera, Vespidae). Insect Soc 50: 82–87.

[77] Stuart RJ, Herbers JM (2000) Nest mate recognition in ants with complex colonies: within- and between population variation. Behav Ecol 11: 676–685.

[78] Carlin NF, Hölldobler B (1986) The kin recognition system of carpenter ants (*Camponotus* spp.). I. Hierarchical cues in small colonies. Behav Ecol Sociobiol 19: 123–134.

[79] Carlin NF, Hölldobler B (1988) Influence of virgin queens on kin recognition in the carpenter ant *Camponotus floridanus* (Hymenoptera : Formicidae). Insect Soc 35: 191–197.

[80] Härtel S, Neumann P, Kryger P, von der Heide C, Moltzer G-J, et al (2006) Infestation levels of *Apis mellifera scutellata* swarms by socially parasitic Cape honeybee workers (*Apis mellifera capensis*). Apidologie 37: 462–470.

[81] Beekman M, Oldroyd BP (2008) When workers disunite: Intraspecific parasitism in eusocial bees. Annu Rev Entomol 53: 19–37. 17600462 10.1146/annurev.ento.53.103106.093515

[82] Chapman NC, Nanork P, Gloag RS, Wattanachaiyingcharoen W, Beekman M, et al (2009) Queenless colonies of the Asian red dwarf honey bee (*Apis florea*) are infiltrated by workers from other queenless colonies. Behav Ecol 65: 817–820.

[83] Stuart RJ (1987) Transient nestmate recognition cues contribute to a multicolonial population structure in the ant, *Leptothorax currispinosus* . Behav Ecol Sociobiol 21: 229–235.

[84] Obin MS, Vander Meer RK (1989) Mechanism of template-label matching in fire ant, *Solenopsis invicta* Buren, nestmate recognition. Anim Behav 38: 430–435.

[85] Lahav S, Soroker V, Vander Meer RK, Hefetz A (1998) Nestmate recognition in the ant *Cataglyphis niger* : do queens matter? Behav Ecol Sociobiol 43: 203–212.

[86] van Zweden JS, Dreier P, d'Ettorre P (2009) Disentangling environmental and heritable nestmate recognition cues in a carpenter ant. J Insect Physiol 55: 158–163. 19041322 10.1016/j.jinsphys.2008.11.001

[87] Bloch G, Borst DW, Huang ZY, Robinson GE, Hefetz A (1996) Effects of social conditions on juvenile hormone mediated reproductive development in *Bombus terrestris* workers. Physiol Entomol 21: 257–267.

[88] Orivel J, Errard C, Dejean A (1997) Ant gardens: interspecific recognition in parabiotic ant species. Behav Ecol Sociobiol 40: 87–93.

[89] Errard C, Ipinza Regla J, Hefetz A (2003) Interspecific recognition in Chilean parabiotic ant species. Insect Soc 50: 268–273.

[90] Grangier J, Le Breton J, Dejean A, Orivel J (2007) Coexistence between *Cyphomyrmex* ants and dominant populations of *Wasmannia auropunctata* . Behav Proc 74: 93–96. 10.1016/j.beproc.2006.10.009 17129678

[91] Liu ZB, Yamane S, Yamamoto H, Wang QC (2000) Nestmate discrimination and cuticular profiles of a temporary parasitic ant *Lasius sp.* and its host *L. fuliginosus* (Hymenoptera, Formicidae). J Ethol 18: 69–73.

[92] Liu ZB, Bagneres AG, Yamane S, Wang QC, Kojima JI (2003) Cuticular hydrocarbons in workers of the slave-making ant *Polyergus samurai* and its slave, *Formica japonica* (Hymenoptera: Formicidae). Entomol Sci 6: 125–133.

