# Supplementary material for: Born in an Alien Nest : How Do Social Parasite Male Offspring Escape from Host Aggression?
Source: PLoS One. 2012 Sep 20;7(9):e43053. doi: 10.1371/journal.pone.0043053 (PMC3447871; doi:10.1371/journal.pone.0043053)
Supplement: Table S1 — Relative abundance (median, rel. %) of the compounds identified in cephalic secretions of Bombus vestalis males. (DOC) [file pone.0043053.s001.doc]

**Table S1:** **Relative abundance (median, rel. %) of the compounds identified in cephalic secretions of *Bombus vestalis* males.**

|  | **1 day old (*n* = 5)** | | **7 days old (*n* = 5)** | |
| --- | --- | --- | --- | --- |
| **Compounds** | ***M*** | ***IQR*** | ***M*** | ***IQR*** |
| Tetradecyl acetate | 0.12 | 0.02 | 0.16 | 0.03 |
| Hexadecen-1-ol | 0.29 | 0.16 | 0.12 | 0.01 |
| Nonadecane | 0.12 | 0.07 | 0.04 | 0.02 |
| Hexadecenyl acetate | 3.97 | 1.44 | 4.50 | 1.22 |
| Hexadecenyl acetate | 0.40 | 0.11 | 0.35 | 0.10 |
| Octadecenal | 1.19 | 0.32 | 0.84 | 0.12 |
| Octadecenal | 0.06 | 0.01 | 0.12 | 0.04 |
| Octadecadien-1-ol | 0.46 | 0.07 | 0.53 | 0.20 |
| Octadecen-1-ol | 0.72 | 0.10 | 0.63 | 0.19 |
| Heneicosane | 0.47 | 0.20 | 0.28 | 0.02 |
| Geranylcitronellol | **38.62** | **2.85** | 31.24 | 9.86 |
| Unidentified C21 | 1.51 | 0.36 | - | - |
| Octadecadienyl acetate | 0.75 | 0.17 | 2.52 | 0.50 |
| Octadecenyl acetate | - | - | 0.55 | 0.22 |
| Icosadienal | 13.46 | 1.40 | 16.80 | 2.89 |
| Icosenal | 1.24 | 0.19 | 2.18 | 0.78 |
| Geranylcitronellyl acetate | 19.39 | 1.41 | **32.55** | **6.42** |
| Icosadienol | 2.12 | 0.45 | 2.22 | 0.26 |
| Icosatrienol | 0.59 | 0.06 | 1.01 | 0.30 |
| Icosenol | 5.77 | 1.83 | 5.12 | 1.77 |
| Unidentified C22 | 0.72 | 0.08 | 1.29 | 0.48 |
| Tricosane | 1.18 | 0.08 | 1.74 | 0.42 |
| Unidentified C22 | 0.37 | 0.18 | 0.33 | 0.14 |
| Eicosadienyl acetate | 0.55 | 0.06 | 1.29 | 0.48 |
| Eicosenyl acetate | 0.30 | 0.10 | 0.37 | 0.06 |
| Unidentified C24 | 0.11 | 0.02 | 0.18 | 0.08 |
| Docosenal | 0.11 | 0.07 | - | - |
| Pentacosene | 0.08 | 0.03 | 0.04 | 0.02 |
| Pentacosane | 1.14 | 0.54 | 0.65 | 0.15 |
| Unidentified C25 | 0.14 | 0.07 | 0.08 | 0.01 |
| Hexacosane | 0.08 | 0.04 | 0.03 | 0.01 |
| Heptacosene | 0.28 | 0.11 | 0.12 | 0.03 |
| Heptacosene | 0.28 | 0.10 | 0.18 | 0.01 |
| Heptacosane | 2.34 | 1.24 | 0.71 | 0.15 |
| Unidentified C26 | 0.35 | 0.17 | 0.09 | 0.00 |
| Octacosane | 0.09 | 0.05 | 0.03 | 0.00 |
| Nonacosene | 0.64 | 0.29 | 0.26 | 0.06 |
| Nonacosene | 0.53 | 0.22 | 0.22 | 0.00 |
| Nonacosane | 1.59 | 0.85 | 0.30 | 0.02 |
| 13-Methylnonacosane | 0.25 | 0.12 | 0.05 | 0.01 |
| Hentriacontene | 0.17 | 0.08 | 0.10 | 0.01 |
| Unidentified C30 | 0.21 | 0.05 | 0.23 | 0.05 |
| Geranylcitronellyl tetradecenoate | 0.10 | 0.03 | 0.09 | 0.02 |
|  |  |  |  |  |

*n* number of males. - the compound is absent in every specimens of the age class. The main compound of each age class is marked in bold. Compounds are listed in the retention order on a DB5-ms column. *M* = Median. *IQR* = interquartile Range (Quartiles 3-1)
