# Supplementary material for: Born in an Alien Nest : How Do Social Parasite Male Offspring Escape from Host Aggression?
Source: PLoS One. 2012 Sep 20;7(9):e43053. doi: 10.1371/journal.pone.0043053 (PMC3447871; doi:10.1371/journal.pone.0043053)
Supplement: Table S2 — Relative abundance (median, rel. %) of the compounds identified in the cephalic secretions of the males of Bombus terrestris ( n = 5), B. pascuorum ( n = 12) and B. vestalis ( n = 5). (DOC) [file pone.0043053.s002.doc]

**Table S2 : Relative abundance (median, rel. %) of the compounds identified in the cephalic secretions of the males of *Bombus terrestris* (*n*=5), *B. pascuorum* (*n*=12) and *B. vestalis* (*n*=5).**

|  | ***B. terrestris*** | | ***B. pascuorum*** | | ***B. vestalis*** | |
| --- | --- | --- | --- | --- | --- | --- |
| **Compounds** | ***M*** | ***IQR*** | ***M*** | ***IQR*** | ***M*** | ***IQR*** |
| Dihydrofarnesal | 2.05 | 1.31 | – | – | – | – |
| Dihydrofarnesol | 78.52 | 44.12 | – | – | – | – |
| Dihydrofarnesyl acetate | 0.09 | 0.06 | – | – | – | – |
| Dihydrofarnesyl dodecanoate | 1.23 | 5.91 | – | – | – | – |
| Dihydrofarnesyl tetradecanoate | 0.03 | 1.1 | – | – | – | – |
| Docosane | – | – | 0.07 | 0.07 | – | – |
| Docosenyl acetate | 0.04 | 0.13 | – | – | – | – |
| Dodecane | – | – | 0.006 | 0.013 | – | – |
| Dodecenyl acetate | – | – | 0.009 | 0.005 | – | – |
| Eicosadienyl acetate | – | – | – | – | 1.29 | 0.48 |
| Eicosane | – | – | 0.003 | 0.004 | – | – |
| Eicosenyl acetate | – | – | – | – | 0.37 | 0.06 |
| Ethyl dodecanoate | 0.45 | 0.68 | – | – | – | – |
| Geranylcitronellal | 1.61 | 2.09 | – | – | – | – |
| Geranylcitronellol | 3.42 | 7.52 | – | – | 31.24 | 9.86 |
| Geranylcitronellyl acetate | – | – | – | – | 32.55 | 6.42 |
| Geranylcitronellyl dodecanoate | 0.01 | 0.16 | – | – | – | – |
| Geranylcitronellyl tetracanoate | 0.05 | 0.18 | – | – | – | – |
| **Heneicosane** | **1.29** | **1.71** | **0.1** | **0.1** | **0.28** | **0.02** |
| Hentriacontane | – | – | 0.008 | 0.01 | – | – |
| Hentriacontene | – | – | 0.03 | 0.025 | 0.10 | 0.01 |
| **Heptacosane** | **0.02** | **0.33** | **0.05** | **0.065** | **0.71** | **0.15** |
| **Heptacosene** | **0.31** | **2.85** | **0.084** | **0.091** | **0.3** | **0.04** |
| Heptadecanal | – | – | 0.087 | 0.012 | – | – |
| Heptanal | – | – | 0.011 | 0.007 | – | – |
| Hexacosane | – | – | 0.006 | 0.007 | 0.03 | 0.01 |
| Hexacosene | 0.03 | 0.41 | 0.005 | 0.007 | – | – |
| Hexadecanal | 0.21 | 0.06 | – | – | – | – |
| Hexadecane | – | – | 0.001 | 0.001 | – | – |
| Hexadecanol | 2.92 | 0.66 | 0.23 | 0.18 | – | – |
| Hexadecenal | 0.22 | 0.10 | 87.25 | 11.79 | – | – |
| Hexadecenol | – | – | 5.51 | 9.66 | 0.12 | 0.01 |
| Hexadecenyl acetate | – | – | 0.014 | 0.017 | 4.85 | 1.32 |
| Hexadecyl acetate | 0.09 | 0.13 | – | – | – | – |
| Icosadienal | – | – | – | – | 16.80 | 2.89 |
| Icosadienol | – | – | – | – | 2.22 | 0.26 |
| Icosatrienol | – | – | – | – | 1.01 | 0.3 |
| Icosenal | – | – | – | – | 2.18 | 0.78 |
| Icosenol | – | – | – | – | 5.12 | 1.77 |
| Methyl hexadecenoate | – | – | 0.004 | 0.005 | – | – |
| Methyl nonacosane | – | – | – | – | 0.05 | 0.01 |
| Nonacosane | – | – | 0.018 | 0.02 | 0.30 | 0.02 |
| **Nonacosene** | **0.06** | **0.46** | **0.027** | **0.027** | **0.48** | **0.06** |
| Nonadecadienal | 0.17 | 0.06 | – | – | – | – |
| Nonadecane | – | – | – | – | 0.04 | 0.02 |
| Octacosane | – | – | – | – | 0.03 | 0.001 |
| Octadecadienal | 0.09 | 0.11 | – | – | – | – |
| Octadecadienol | 1.98 | 3.36 | – | – | 0.53 | 0.2 |
| Octadecadienyl acetate | 0.71 | 1.09 | – | – | 2.52 | 0.5 |
| Octadecatrienal | 0.13 | 0.05 | – | – | – | – |
| Octadecenal | – | – | – | – | 0.96 | 0.16 |
| Octadecenol | – | – | 0.84 | 0.4 | 0.63 | 0.19 |
| Octadecenyl acetate | – | – | – | – | 0.55 | 0.22 |
| **Pentacosane** | **0.23** | **1.58** | **0.46** | **0.53** | **0.65** | **0.15** |
| **Pentacosene** | **0.06** | **0.96** | **0.41** | **0.47** | **0.04** | **0.02** |
| Pentadecane | – | – | 0.001 | 0.001 | – | – |
| Pentadecenal | – | – | 0.087 | 0.046 | – | – |
| Pentadecene | – | – | 0.02 | 0.012 | – | – |
| Pentadecenol | – | – | 0.16 | 0.17 | – | – |
| Tetracosane | – | – | 0.028 | 0.038 | – | – |
| Tetracosene | – | – | 0.007 | 0.009 | – | – |
| Tetradecanal | 0.86 | 0.29 | – | – | – | – |
| Tetradecanoic acid | 0.14 | 0.25 | – | – | – | – |
| Tetradecanol | – | – | 0.114 | 0.084 | – | – |
| Tetradecenol | – | – | 0.081 | 0.087 | – | – |
| Tetradecyl acetate | – | – | – | – | 0.16 | 0.03 |
| **Tricosane** | **1.35** | **8.21** | **1.82** | **1.8** | **1.74** | **0.42** |
| Tricosene | 0.27 | 1.31 | 0.042 | 0.045 | – | – |
| Tridecane | – | – | 0.008 | 0.016 | – | – |
| Undecane | – | – | 0.002 | 0.004 | – | – |
| Undecenal | – | – | 0.007 | 0.004 | – | – |

The compounds present in the three species are marked in bold. *M* = Median. *IQR* = interquartile Range (Quartiles 3-1)
